# Diet Quality among Pre-Adolescent African American Girls in a Randomized Controlled Obesity Prevention Intervention Trial

**DOI:** 10.3390/nu15122716

**Published:** 2023-06-12

**Authors:** Debbe Thompson, Yiming Mirabile, Noemi Islam, Chishinga Callender, Salma M. A. Musaad, Julie Miranda, Jennette P. Moreno, Jayna M. Dave, Tom Baranowski

**Affiliations:** USDA/ARS Children’s Nutrition Research Center, Department of Pediatrics, Baylor College of Medicine, 1100 Bates Street, Houston, TX 77030, USA

**Keywords:** child, Black or African American, healthy diet

## Abstract

Consuming an unhealthy diet increases health risks. This study assessed the impact of a culturally adapted behaviorally innovative obesity prevention intervention (*The Butterfly Girls and the Quest for Founder’s Rock*) on diet quality in pre-adolescent non-Hispanic Black/African American girls. The RCT consisted of three groups (experimental, comparison, and waitlist control); block randomization allocated participants to each group. The two treatment groups varied in terms of whether or not they set goals. Data were collected at baseline (prior to receiving the intervention), post 1 (3 months post-baseline), and post 2 (6 months post-baseline). Two dietitian-assisted 24 h dietary recalls were collected at each timepoint. Healthy Eating Index 2015 (HEI-2015) was used to determine diet quality. A total of 361 families were recruited; 342 completed baseline data collection. No significant differences in overall HEI score or component scores were observed. To attain more equitable health outcomes, future efforts to promote dietary intake change among at-risk children should explore other behavior change procedures and employ more child-friendly dietary assessment methods.

## 1. Introduction

Childhood obesity remains a major public health issue [1], particularly among children of minority descent [2]. A child obesity prevention online intervention, designed to ensure cultural relevance and developmental appropriateness [3] while promoting healthy diet and physical activity, may provide a convenient and accessible method for impacting child obesity.

During the height of the COVID-19 pandemic, child obesity in the United States (US) increased from 19.3% in August 2019 to 22.4% in August 2020, an all-time high [1]. In the US, obesity prevalence is unequally distributed, with non-Hispanic Black/African American (NH B/AA) 2–19-year-old youth being more likely to have obesity compared to their non-Hispanic White peers (16.1% vs. 24.2%) [2].

Obesity steadily increases throughout childhood and adolescence. Among children in the US, 13.4% of 2–5-year-olds, 20.3% of 6–11-year-olds, and 21.2% of 12–19-year-olds are reported to have obesity [2]. Obesity follows one into adulthood, with adolescents having obesity more likely to become adults with obesity [4,5,6]. Once established, obesity is difficult to treat [7,8,9] or reverse [10,11], strengthening the case for primary prevention among children [12], a growing worldwide public health priority [13].

Consuming an unhealthy diet increases the risks of obesity, diet-related chronic diseases (e.g., cardiovascular disease, diabetes, and some cancers [14]) and earlier mortality [15]. Most US children do not meet dietary guidelines [16]. Findings from a study with a nationally representative sample of 2–19-year-old children in the US revealed that from 2001 to 2018, junk food consumption (e.g., sweet bakery products, crackers, and candy) was high and stable over time [17], thereby emphasizing the importance of early intervention to enhance dietary intake. However, most interventions to improve diet among 6–12-year-old children have had little to no effect [18]. Thus, innovative interventions that engage and motivate this age group are needed.

Racial disparities exist in dietary patterns, which may partially explain the increased prevalence of overweight and obesity in minority children. Compared to non-Hispanic White children, NH B/AA children in the Special Supplemental Nutrition Program for Women, Infants, and Children (WIC) had lower calcium, vitamin D, and total dairy intakes and higher sodium intakes [19]. Further, diets of NH B/AA youth were lower in fruit, vegetables, and low-fat dairy products when compared to those of non-Hispanic White children [20,21,22]. These dietary patterns have been associated with increased body weight and obesity risk [23].

Although the relationship between diet and obesity is known to be associated with sustained energy imbalance, no consensus exists on the best approach to assess the association between diet and childhood obesity [24]. Diet quality provides an easy-to-track measure of diet healthfulness. Diet quality, broadly defined as a comparison of one’s dietary intake to dietary guidelines, has gained increasing attention as a predictor of childhood obesity [24,25]. Higher diet quality scores have also been associated with lower risk of chronic diseases [26]. Measuring diet quality among children at greater risk of obesity, such as NH B/AA children, may provide valuable insights into the effectiveness of methods for reducing obesity risk [27].

Healthy Eating Index 2015 (HEI-2015) measures diet quality as assessed via determining whether or not an individual’s dietary intake aligns with the 2015–2020 Dietary Guidelines for Americans [28]. HEI-2015 includes 13 components with a maximum total score of 100, indicating perfect compliance with the Dietary Guidelines. It assesses overall intake of calories and energy density, both of which have been associated with obesity [28]. Thus, HEI may be a valuable tool for assessing the complex relationships between dietary intake and obesity in children [25,29]. Furthermore, the key food groups and nutrients in the HEI are commonly targeted for intervention and are known predictors of chronic diseases [30].

Diet quality in the US is below that of the recommendations [31,32]. Even though modest improvements were observed among 2–18-year-old youth (from 1999 to 2016), more than half were classified as having poor-quality diets [32]. Similarly, in a nationally representative sample of 2–18-year-old youth (n = 9000), overall diet quality measured using HEI was 54.9 (out of 100) [33]. NH B/AA children had lower scores (52.6) than Mexican American (57.0) or non-Hispanic White children (54.2), while HEI scores did not differ according to child sex or poverty status [33]. Another nationally representative sample reported lower diet quality among NH B/AA 2–18-year-old youth (48.4) compared to that of their peers (50.9) (n = 38,497) [34], supporting the need for culturally sensitive interventions to address this disparity.

An internet-based intervention provides a method that can be easily accessed by parents and children at their most convenient times using digital devices, such as a computer or smartphone. Effective digital interventions can also be scaled to reach larger audiences at little additional cost. While NH B/AA families have been shown to regularly access the internet [35], an effective intervention should be culturally tailored to their needs and interests, most often by involving key stakeholder groups in their development [36]. Given the lack of effectiveness of most dietary change [37] and obesity prevention interventions [18] with this age group, it is important for interventions to incorporate innovative intervention techniques. The use of a story/narrative, implementation intentions and schemas offer potentially effective techniques.

Engagement is a characteristic of whether or not a participant is paying attention to and involved with the intervention [38]. If not engaged, the participant will be less likely to pay attention to the messages in the intervention and thereby not be expected to benefit from participation. The use of a story or narrative can obtain [39] and maintain [40] engagement. Story episodes, embedded in multiple sessions of an intervention, benefit from ending with a cliff-hanger to encourage participants to return to see the continuation of the story [41].

Goal setting is a common behavior change technique in behavioral interventions [42]. Implementation intentions is a goal setting procedure wherein very specific goals and plans for attaining them are set, specifying what, where, when and how the behavior will be implemented [43]. Implementation intentions were shown to effectively produce longer term dietary change effects in our previous research with pre-adolescent children [44]. Schemas are aspects of a person’s self-concept; people tend to act consistently with their self-concept [45,46]. Schemas associate characteristics of a child’s behavior (e.g., eating a certain number of fruit and vegetables) with characteristics of the environment (e.g., at lunch at school) which signal or evoke the relevant behavior. Schemas have been successfully employed in dietary change interventions with children [47].

Using a three-group randomized controlled trial (RCT) design, the current study encouraged a healthy diet to pre-adolescent NH B/AA girls and their parent. The purpose of this paper is to report changes in diet quality over time.

## 2. Materials and Methods

### 2.1. Research Overview and Intervention Description

Although described in detail elsewhere [3], the intervention, named the *Butterfly Girls and the Quest for Founder’s Rock* (BFG), is briefly described here. BFG was designed to prevent obesity among 8–10-year-old NH B/AA girls. The RCT consisted of three groups (experimental, comparison, and waitlist control). Block randomization was used to allocate participants to the three groups. The block strategy balances an equal number of participants in each group. The computer algorithm written in SAS (version 9.3, 2012, Cary, NC, USA) was used to perform block randomization using the PROC PLAN procedure and the computer system clock for the random seed [48]. Data were collected at baseline (prior to receiving the intervention), post 1 (after intervention completion—i.e., approximately 3 months post-baseline), and post 2 (approximately 6 months post-baseline). The intervention, delivered online, consisted of eight animated episodes promoting five servings of fruit and vegetables, five glasses of water, and 60 min of physical activity per day. After viewing each episode, girls in the experimental group set diet, physical activity, or water goals for the following week. Schemas [49] were developed to facilitate goal setting and to make it easier and more appealing. For example, girls could be a Breakfast Builder, Lunch Leader, Dynamite Diner, or Star Snacker based on which meal or snack they wanted to eat more fruit and vegetables from (e.g., Breakfast Builders ate two fruit and vegetables at breakfast and one each at lunch, dinner, and snack time. Alternatively, a Star Snacker ate one fruit or vegetable at meals and two of for the snack). Similar schemas were developed for physical activity and water. Between episodes, girls were provided a tracking sheet to monitor their goal attainment. At the beginning of episodes 2–8, girls reported their goal attainment and received feedback tailored to their level of goal attainment (e.g., if they met all 3 challenges of consuming 5 a day, they received the message “Great job! You get better and better each week. You’re an awesome friend.”; if they met none of their challenges, they received the message “Meeting your challenges can be hard, but with a plan and a little hard work, you can do it! Check out the fun page for ideas.”).

Girls in the experimental group received the full intervention—i.e., they watched eight animated BFG episodes, set diet and physical activity goals, tracked and reported goal progress, and received feedback tailored to goal attainment. Girls randomized to the comparison group received only the animated stories, while girls randomized to the waitlist control group received the experimental intervention after completing all three data collection timepoints (baseline, post 1, and post 2). This design provided tests of the story alone, and of the added goal setting intervention and feedback.

The BFG program was adapted from an earlier pilot intervention, *Fun, Food, & Fitness*, consisting of a summer day camp, followed by an internet component encouraging the continued use of the behavior change strategies learned in the summer day camp [50]. Due to low participation in the internet component, a later study tested the internet component as a stand-alone intervention (*Food, Fun, and Fitness Internet Program for Girls*) [51].

Because the initial intervention was developed and tested over a decade earlier, community-engaged research [52,53] was conducted with three groups of stakeholders, 8–10-year-old NH B/AA girls, their parents/caregivers, and community representatives, to understand contemporary perceptions of and expectations for an intervention promoting obesity prevention behaviors to young NH B/AA girls. Guided by a theoretical framework comprising social cognitive theory [54] and the elaboration likelihood model [55], stakeholder feedback informed the content, structure, and format of the updated intervention (BFG). Modifications included updated character representations and personality profiles; an action adventure storyline told from the second person perspective (the original program featured a series of loosely connected vignettes); interactivity; a new vocal track voiced by professional actors; a customized music score; and updated graphics. To further ensure the intervention was culturally sensitive and appropriate, an award-winning NH B/AA female playwright who was both a mother and grandmother authored the storyline.

### 2.2. Participants

Child inclusionary criteria included being an 8–10-year-old NH B/AA girl with a parent or legal guardian willing to participate in data collection, internet access, and a personal email address. Exclusionary criteria included having mental, physical, or medical conditions limiting their ability to participate in data collection activities or taking medications that impacted appetite, diet, and physical activity.

Parent inclusionary criteria included having a girl participating in the program, willingness to participate in data collection, having internet access, and having a personal email address; exclusionary criteria included physical or mental restrictions limiting their ability to participate in data collection activities. Only one child and parent per family could participate in the intervention.

### 2.3. Sample Size and Power

The primary outcome measure was the body mass index (BMI) percentile. The estimated sample size (SS) was based on the number of participants needed to detect a significant intervention group by the time interaction effect on the BMI percentile. Data from the *Fun, Food, & Fitness* study of 8–10-year-old NH B/AA girls yielded a baseline BMI percentile of 89.4 ± 14.7 (n = 35) [50]. Given a 0.05 level of significance, a pooled standard deviation of 14.7, and autocorrelations of 0.50, a sample of 324 participants would adequately power (≥80%) the study to detect a small (SEF; standardized effect size, f = 0.13) group by the time interaction, i.e., a 4.1% increase in the BMI percentile over time in the comparison and wait list control groups while the treatment group BMI percentile would remain stable. Given the needed final sample of 324 participants allowing for 20% attrition, the recruitment goal was 390 participants.

### 2.4. Recruitment

Families were recruited using the volunteer database at the Children’s Nutrition Research Center (CNRC), recruitment announcements on websites and in newsletters (e.g., CNRC, Baylor College of Medicine, Texas Children’s Hospital), posting flyers in community locations (e.g., libraries), mailing flyers to community members and organizations, community events (e.g., health fairs), local radio advertisements, and word of mouth. A rolling recruitment method was utilized. Recruitment started in November 2012 and ended in October 2014. Written informed parental consent and child assent were received prior to study participation.

### 2.5. Data Collection Methods

Data were collected from both parents and girls at the three data collection time points (baseline, post 1, and post 2). Both parents and girls completed online self-reported surveys. The surveys were hosted on a secure, password-protected website. Parents and girls received separate links and protected passwords to complete the surveys. Girls also completed two dietitian-conducted 24 h dietary recalls (24-HDR) over the phone. Parents and girls each received a USD 40, USD 50, and USD 60 check after the completion of baseline, post 1, and post 2 data collection, respectively.

### 2.6. Measures—Demographics and Household Characteristics

At baseline data collection, parents of the girls participating in the study completed the demographic and household characteristics questionnaire. These included the number of children and adults in the home, number of evening meals the family eats together at home during weekdays, the number of days the child eats at restaurants, the presence or lack thereof of a TV in the room where the child sleeps, child race/ethnicity and intake of the National School Lunch Program’s (NSLP) free/reduced-price school lunch, and parental age, race/ethnicity, education, and income. The number of hours of reported media use in a typical week was calculated based on parents’ report of the number of hours the child spent watching TV, DVDs and videos, using a computer for something other than activities related to school, and playing video games on handheld devices (e.g., like Xbox, Wii, PlayStation, iPod).

### 2.7. Child Dietary Intake Assessment

Dietary intake assessment was carried out via telephone interviews conducted by dietitians (interviewer-assisted 24-HDR) using Nutrient Data System for Research (NDSR) version 2012 from the University of Minnesota. NDSR relies on the AM/PM and multiple-pass methods and requires extensive probing. Interviewers received training and were certified on data collection methods as well as quality assurance by a senior dietitian which was highly experienced in this method. Child participants completed a total of six 24-HDR: two at baseline, two at post 1 (about 3 months after baseline) and two at post 2 (approximately 6 months post-baseline). The two interviews per period captured dietary patterns on weekdays (school days) and on weekends (non-school days). Each interview represented the complete 24 h period (from midnight to midnight) of the day preceding the day of the interview. Others have collected 24-HDR with children as young as 8 years old [56].

In addition to obtaining detailed information on foods/beverages consumed, interviewers inquired about “activity while eating” and “eating companions” for each of the reported eating occasions. A Food Amount Booklet (FAB) was provided to facilitate portion size estimation. Parents did not participate in the interview, but whenever necessary, girls were allowed to obtain complementary information from the food preparer before completing each telephone interview. For quality assurance purposes, the parent was contacted post-interview to inquire about plausibility if the dietitian questioned the type or amount of a food reported by the child or if food or calorie consumption was suspiciously low or high.

### 2.8. HEI Calculation

All HEI component calculations were generated following the HEI-2015 scoring algorithm outlined by the University of Minnesota, Nutrition Coordinating Center (NCC) [57]. There are 13 components that make up the HEI-2015 [58]. Nine adequacy components include Total Fruits, Whole Fruits, Total Vegetables, Greens and Beans, Whole Grains, Dairy, Total Protein Foods, Seafood and Plant Proteins, and Fatty Acids. Four moderation components (i.e., foods which should be consumed in limited amounts) include refined grains, sodium, added sugars, and saturated fats. The values for each component were calculated based on the summation of the measured food groups. Adequacy components for the study, based on the 24-HDR, consisted of the total fruit juices and avocados (e.g., citrus juices, fruit juices excluding citrus juices, fruit, and avocados); whole fruit (i.e., the total fruit); total vegetables (e.g., dark-green and deep-yellow vegetables, tomatoes, potatoes, and legumes); greens and beans (i.e., dark-green vegetables and legumes); whole grains (i.e., grains, flour, bread, pasta and cereal); dairy (i.e., all milk types, non-dairy milk, flavored milk, cheese, yogurt, and dairy-based meal replacements); total protein foods (i.e., beef, veal, lamb, pork, poultry, fish, eggs, nuts and seeds, meat alternatives, and legumes); seafood and plant proteins (i.e., fish, shellfish, nuts and seeds, meat alternatives, and legumes); and fatty acids (i.e., the ratio of the sum of total monounsaturated and total polyunsaturated fatty acids to total saturated fatty acids). Moderation components consisted of refined grains (e.g., refined grains, cakes, cookies, flour, dry mixes and pasta with some whole grain, corn muffins, and tortillas), sodium, added sugars and saturated fats. Moderation components were provided by NDSR Intake Properties Totals File.

Total HEI score is a combination of all the above components. Total energy intake (kcal) was used to scale each component. Following the recommended variable adjustments from the NCC, the total HEI score was calculated taking the sum of all adjusted components.

### 2.9. Data Analysis

This is a secondary analysis of data from a study previously reporting no differences in BMI by group [59]. Data were descriptively summarized as mean ± standard deviation (SD) or n (%). Baseline demographic and other characteristics were summarized and compared across the randomized groups (Table 1). HEI-2015 total and component scores were summarized and compared across randomization groups and time using the Kruskal–Wallis test.

To determine how raw HEI scores differed over time across intervention groups (i.e., experimental and comparison groups), the association of the intervention with the outcome of HEI scores was tested using the generalized estimating equation model (GEE) specifying an exchangeable correlation structure for the repeated time measurements. A log-normal outcome was specified in the model with a log link function. This was accomplished by specifying the outcome distribution during the modeling process and allowed the response variable to be modeled as a random variable with a Poisson distribution. The randomized group, time and interactions were tested, with the latter serving as the parameter for the effect of the randomized group on the change in HEI scores over time. Since the 24-HDR assessments were conducted on a weekend and weekday, day type (weekend and weekday) was included as a fixed effect. A significant randomized group × time interaction warranted pairwise comparisons between the time points with adjustment for pairwise comparisons using Tukey’s method. To determine which variables to include as covariates in the models, general linear modeling with backward selection (GLMSELECT procedure in SAS) was performed to test the association of the HEI scores as outcomes with the demographic variables. The relationships between the raw HEI scores with covariates were also assessed using the correlation coefficient (Spearman’s rho) for continuous variables, and the Wilcoxon or Kruskall–Wallis test for categorical variables. To keep the models consistent for comparison purposes, a covariate was included in all models if it was associated (*p* < 0.01) with one or more HEI scores. Using this approach, the models were adjusted for the intake of free/reduced-price lunch, hours of reported media use in a typical week, parental education, and whether or not there was a TV in the room where the child sleeps as covariates. Significance tests were determined using type III tests and parameter estimates (beta coefficients). The significance level was lowered to 0.01 to adjust for multiple testing. Analyses were conducted using SAS version 9.4 (SAS Institute, Inc., Cary, NC, USA), R 4.2.2 (2022-10-31 ucrt) in the RStudio integrated development environment (version 2022.12.0.353), tidyverse version 1.3.2 [60] and rstatix package version 0.7.1 [61].

Given the observed associations of the raw HEI scores with the covariates selected for modeling, adjusted HEI scores were calculated to present more precise estimates (Table 2). Adjusted HEI scores were generated using general linear models of the HEI score as the outcome with day type and the covariates selected in the GEE models as independent variables. Since no significant differences in mean raw and adjusted HEI scores were observed, only adjusted HEI scores are presented. Since no significant differences were detected across experimental groups, by group or by time, a table describing group values by time is not presented.

## 3. Results

A total of 361 families were recruited. Of these, 342 completed the baseline data collection. Most families (70.2%) had two or three children and one adult other than the participating parent (53.2%) living at home. The number of weekday evening meals eaten at home varied from two to five (88.3%), while most children (87.7%) ate at least one meal per week at a restaurant. Most parents (65.2%) had a college degree or higher education but had an annual income of USD 60,000 (60.5%) or lower. Somewhat more children (53.2%) received a free/reduced-price lunch at school (indicating lower income), while most children (64.3%) had a TV in the room where they slept.

A significant negative correlation was found between the HEI whole fruit component score and the hours of reported media at baseline (r = −0.2178, *p* < 0.0001). Intake of free/reduced-priced lunch was associated with the total dairy HEI (*p* = 0.0009) and the sodium components (*p* = 0.004). Table 2 descriptively presents the adjusted HEI scores for each of the randomized groups at baseline. No significant differences were observed.

No significant effects were observed for the randomized group x time interaction or for group or time effects with any HEI score using the GEE models. Day type was found to be significant for the HEI total score and each component with higher quality for weekdays than weekends. Associations were observed among the covariates tested. Intake of free/reduced-price lunch was significantly associated with a small increase in the HEI total score (beta coefficient (SE) = 1.0350 (0.0001), *p* = 0.0004), as well as HEI scores for vegetables (1.1449 (0.0026), *p* = 0.0024), dairy (1.1036 (0.0018), *p* = 0.0097), and added sugars (1.0393 (8.6 × 10^−5^), *p* < 0.0001). Hours of child’s media use was significantly associated with a decrease in the HEI total score (0.9976 (8.06 × 10^−7^), *p* = 0.0057). Parental education (college or higher vs. lower levels of education) was significantly associated with higher HEI scores for whole fruits (1.1841 (0.0055), *p* = 0.007).

## 4. Discussion

To our surprise this adequately powered RCT testing a culturally adapted internet intervention with innovative behavior change procedures (i.e., story or narrative, implementation intentions and dietary self-schemas) did not have the desired or expected effects on the quality of the children’s dietary intake. This is consistent with the findings of other children’s dietary intake change [37] and obesity prevention [18] interventions, with no obvious explanation for the lack of effectiveness. The high retention and exposure rate (most girls in the two intervention groups watched all eight episodes) [62] yielded the power to adequately test the effect.

Children were from households with generally well-educated parents who had lower incomes. The families tended to eat at fast food or buffet-type restaurants once or more times per week, where families have less control of the foods served and the methods of preparation. In the overall models controlling for demographics, weekdays were associated with higher diet quality for every component score. This suggests that family meals at home enhanced diet quality, as opposed to meals eaten on weekends when eating out was more likely. Others have found a positive association between home food preparation and diet quality [63]. Child participation in free/reduced-price school lunch was associated with higher total diet quality and with higher component scores for vegetables, dairy as well as sugar, indicating that school-provided lunches enhanced diet quality except for sugar intake. A recent systematic review of universal school meals reported that most studies reported a desirable relationship between free school lunch and child diet quality [64]. The hours of child screen media use were negatively associated with total diet quality, which is consistent with the findings of other research [65], suggesting that exposure to commercials influenced child dietary intake, thereby detracting from diet quality. Consistently with the findings of previous research [32,66], parental higher education was associated with higher child diet quality; this suggests that more well-educated parents were both more well-informed about a healthy diet and how to encourage their child to consume one.

The tailoring of the intervention to one ethnic group should have simplified the design of the intervention. This should have avoided giving possible conflicting messages to the children, while the involvement of stakeholders should have ensured the use of culturally appropriate normative messages and procedures [36], thereby enhancing diet quality. Future research is needed to examine this outcome in more detail and understand the reasons the expected outcome did not occur. It could be that, given the heterogeneity of the sample, different normative messages were needed that were tailored to the key social determinants of health that are likely to impact diet quality (e.g., household income and food security) [32].

The age of the participants (8–10 years old) has been associated with lower accuracy in self-completed dietary intake self-reporting [67]. The use of interviewer-conducted 24-HDR with the state-of-the-art NDSR data collection software should have minimized these problems. Using chest-worn-camera images of dietary intake [68] may hold some promise for minimizing future reporting errors. Further support for this can be obtained from reviewing the post-intervention reactions to the intervention. While both parents and girls in the intervention groups reported favorable reactions to the program, a parent mentioned that the dietary recalls were burdensome for her daughter [62]. Anecdotal information shared by dietitians during data collection support that the dietary recalls were difficult for the girls, supporting other reports that children this age have difficulty accurately reporting dietary intake [69]. Intrusions and omissions of foods during recalls likely impacted accuracy and lend support to the need to find more objective, less burdensome data collection methods for pre-adolescent children.

The HEI may have limited variability [70]. For example, on a scale from 0 to 100, the HEI total score had a standard deviation varying from 1.01 to 1.2, perhaps indicating intervention resistance. This might account for some of the lack of impact on the overall diet quality, but not for that on the sub-scores. Changes have been detected in some of these food groups [47].

The HEI-2015 scores in our sample (total HEI-2015 = 52.77 to 53) were somewhat lower than those detected in Spain (total HEI-2015 = 59.2 +/− 8.5) [71]. Notice the substantially larger standard deviation in Spain. Our sample had larger mean values for fatty acid ratio, sodium, refined grains and saturated fats with near-equal values of added sugars. HEI-2015 scores in our study sample were also lower than those detected among US Hispanic (HEI-2015 = 56.1) children, but were similar among NH B/AA children in the US (HEI-2015 = 53.3) that apparently had standard deviations close to those we detected. Alternatively, the HEI-2015 score in our study sample was substantially higher than that among obese Turkish children (HEI-2015 = 47.87) [72]. A nationally representative US sample indicated a variability in total HEI-2015 scores over time from 48.7 in 1999–2000 to 51.61 in 2017–2018 [73]. It is not clear the extent to which any difference in methods may account for the differences in values. Systematic reviews and additional research are needed in regard to the HEI-2015 among children. 

The primary strengths of this study included the adequately powered sample, strict block randomization design, theory-specified intervention techniques informed by stakeholders, state-of-the-art measures, sophisticated statistical analyses, high engagement, and high retention rates. The limitations include the possible inaccuracy of the reported dietary intake in this age group (which should have been minimized using dietitian conducted 24-HDR with the NDSR) and the possible representational biases associated with the use of a non-randomly selected sample. Further, the study was conducted from November 2012–May 2015. Therefore, some of the participant characteristics, such as type, availability, use of technology and other screentime characteristics (i.e., television in their bedroom) may be different from those of a more contemporary sample.

## 5. Conclusions

In this more well-educated but lower-income sample of NH B/AA children and parents, a RCT testing a culturally adapted internet intervention with innovative behavior change procedures did not have the desired or expected effects on children’s dietary intake overall, although differences were observed among those with certain characteristics, such as children receiving free/reduced-price lunch at school and screen media use. Future research is needed to further investigate these relationships. The reasons for the lack of overall effectiveness are not clear. Future efforts to promote dietary intake change among NH B/AA children should explore other behavior change procedures and employ less error-prone dietary intake assessment methods. Poor dietary intake and obesity remain frequently addressed, but unresolved, public health problems in need of intense solution-focused research efforts.

## 6. Implications

This research demonstrates that a thoughtfully designed intervention using innovative behavioral methods did not achieve the intended outcomes in terms of dietary changes. There are two primary implications resulting from this research. First, it suggests that more foundational work is needed with pre-adolescent NH B/AA girls to identify procedures and delivery methods that are more likely to lead to behavior change. Second, it suggests that the dietary assessment method, 24-HR dietitian-assisted dietary recalls, was not appropriate for this age group and that more passive assessment methods, such as those using technology, may provide a more accurate assessment of dietary intake. These findings imply that more work is needed to identify effective ways to modify behavior and achieve more equitable outcomes in diet and diet-related chronic diseases in under-represented children. This is an urgent public health priority and speaks to the importance of publishing non-significant findings so that we can learn from one another in our collective pursuit to advance knowledge and attain health equity.

## Figures and Tables

**Table 1:** Demographics and other characteristics in the full sample.

Demographics and Other Characteristics	Randomized Group						N	(%)
	Experimental (Story and Goal Setting)		Comparison (Story Only)		Wait List Control			
	N	(%)	N	(%)	N	(%)		
All	114	100.00	114	100.00	114	100.00	342	100.00
Number of children under the age of 18 living at home								
0 to 1	24	21.05	19	16.67	13	11.40	56	16.37
2 to 3	76	66.67	84	73.68	80	70.18	240	70.18
>=4	14	12.28	11	9.65	21	18.42	46	13.45
Not including parent participating in the study, number of adults living at home								
0	24	21.05	17	14.91	19	16.67	60	17.54
1	57	50.00	62	54.39	63	55.26	182	53.22
2 to 3	32	28.07	26	22.81	32	28.07	90	26.32
>=4	1	0.88	9	7.89	.	.	10	2.92
Number of evening meals family eats at home during the week								
0 to 1	16	14.04	10	8.77	14	12.28	40	11.70
2 to 3	44	38.60	56	49.12	39	34.21	139	40.64
4 to 5	54	47.37	48	42.11	61	53.51	163	47.66
Number of days/week the child eats at restaurants (e.g., fast food and buffets)								
0	12	10.53	14	12.28	16	14.04	42	12.28
1	33	28.95	53	46.49	43	37.72	129	37.72
2	37	32.46	22	19.30	35	30.70	94	27.49
>=3	32	28.07	25	21.93	20	17.54	77	22.51
Parental education level								
College or higher	78	68.42	70	61.40	75	65.79	223	65.20
Below college-level	36	31.58	44	38.60	39	34.21	119	34.80
Annual household income								
<=USD 61,000	61	53.51	76	66.67	70	61.40	207	60.53
>USD 61,000	53	46.49	38	33.33	44	38.60	135	39.47
NSLP participation								
Free/reduced-price lunch	54	47.37	65	57.02	63	55.26	182	53.22
Full-priced lunch/other	60	52.63	49	42.98	51	44.74	160	46.78
TV in room where child sleeps								
Yes	74	64.91	77	67.54	69	60.53	220	64.33
No	40	35.09	37	32.46	45	39.47	122	35.67

**Table 2:** Descriptive statistics of adjusted HEI scores at baseline.

	Randomized Group								
	Experimental (Story and Goal Setting)			Comparison (Story Only)			Wait List Control		
	N	Mean	Std	N	Mean	Std	N	Mean	Std
Total Score	107	52.77	1.20	103	52.95	1.01	109	53.00	1.05
Total Vegetables (0–5)	107	1.18	0.12	103	1.18	0.09	109	1.20	0.11
Greens and Beans (0–5)	107	0.81	0.12	103	0.81	0.11	109	0.82	0.11
Total Fruit (0–5)	107	1.51	0.15	103	1.50	0.10	109	1.53	0.14
Whole Fruit (0–5)	107	1.41	0.23	103	1.41	0.18	109	1.44	0.20
Whole Grains (0–10)	107	2.08	0.21	103	2.09	0.12	109	2.10	0.19
Dairy (0–10)	107	3.06	0.36	103	3.07	0.23	109	3.10	0.33
Total Protein Foods (0–5)	107	2.63	0.16	103	2.61	0.08	109	2.63	0.15
Seafood and Plant Protein (0–5)	107	1.05	0.12	103	1.05	0.11	109	1.06	0.11
Fatty Acid Ratio (0–10)	107	5.19	0.22	103	5.22	0.22	109	5.20	0.24
Sodium (0–10)	107	8.76	0.26	103	8.76	0.18	109	8.75	0.24
Refined Grains (0–10)	107	8.81	0.17	103	8.85	0.11	109	8.82	0.19
Added Sugars (0–10)	107	8.91	0.23	103	8.94	0.18	109	8.94	0.22
Saturated Fats (0–10)	107	7.36	0.39	103	7.45	0.28	109	7.42	0.41

Notes: Adjusted HEI scores were generated using general linear model adjusting for day type, parental education level, free/reduced-price lunch, TV in room where child sleeps, and hours in typical week of child’s media use, then averaged across day type (weekday and weekend). No significant differences across randomization groups were observed. Missing values per randomized group: n = 7 (story and goal setting), n = 11 (story only), and n = 5 (waitlist control).

## Data Availability

The datasets generated and/or analyzed during the current study are not publicly available due to concerns regarding privacy but select data are available from the corresponding author upon reasonable request.
